# Fetal giant right cervical cyst causing severe tracheal compression: A case report

**DOI:** 10.1097/MD.0000000000016670

**Published:** 2019-08-02

**Authors:** Yanming Kang, Yushan Ma, Xiaoqin Jiang, Xuemei Lin, Fumin Zhao

**Affiliations:** a Department of anesthesiology; b Key Laboratory of Birth Defects and Related Diseases of Women and Children (Sichuan University), Ministry of Education; c Radiology Department of Radiology, West China Second University Hospital, Sichuan University, Chengdu, Sichuan Provence, China.

**Keywords:** fetal giant cervical cyst, general anesthesia, neonatal intralesional sclerotherapy, tracheal compression

## Abstract

**Rationale:**

Fetal giant cervical cyst (FGCC) is a rare congenital anomaly. Sometimes FGCC may extend into the mediastinum, and result in severe tracheal compression, which is a life-threatening event at birth.

**Patient concerns:**

We present a rare case of FGCC, which extended from the right neck into the superior mediastinum, and resulted in severe tracheal compression.

**Diagnoses:**

An FGCC was observed by ultrasonography and magnetic resonance imaging (MRI) at 27+4 weeks’ gestation (WG). Fetal MRI at 35+1 WG showed that the FGCC was 3.3 × 8.2 × 7.5 cm and extended from the right neck into the superior mediastinum. Severe tracheal compression was observed and the inside diameter of the narrowest section of tracheostenosis appeared thread-like and measured only 0.1 cm.

**Interventions:**

Cervical cyst reduction was performed prenatally under ultrasound guidance to alleviate the tracheal compression and maximize the chance of fetal survival 2 days before birth. At 36+3 WG, cesarean section was performed, and a female neonate was immediately delivered and intubated (3.5-mm tube) by an experienced anesthesiologist. Neonatal intralesional sclerotherapy and cystic component aspiration as guided by digital subtraction angiography were performed under general anesthesia. Anesthesia was maintained only with sevoflurane 3% in 2 L/min oxygen. Extubation was performed soon after surgery.

**Outcome:**

The neonate recovered uneventfully and was discharged 2 days postoperatively. After 140 days of follow-up, the neonate had recovered completely.

**Lessons:**

If an FGCC is suspected by abdominal ultrasound, a fetal MRI is recommended to assess the severity of tracheal compression before birth, if feasible. An anesthesiologist should assess the risk of intubation failure at birth according to those results. If fetal severe tracheal compression is detected and it may result in inability of intubation at birth, prenatal cervical cyst reduction under ultrasound guidance may be effective for alleviating tracheal compression at birth, if feasible. This could maximize the chance of fetal survival. Improvement of fetal short- and long-term outcomes is important.

## 1 Introduction

Fetal tumors are rare congenital anomalies, with an estimated incidence of 1/12,000 to 1/30,000 births.^[1]^ Among them, fetal cervical cysts^[2,3]^, such as lymphangioma,^[4]^ represent an important group of tumors. Fetal cervical cysts are soft, benign, and occur by fluid-filled cystic dilation, and are typically found in the posterior triangle of the neck. A few of these cysts may extend into the axilla or mediastinum.^[5]^ If these cysts extend into the mediastinum, a severe fetal giant cervical cyst (FGCC) may result in severe tracheal compression, which is a life-threatening event at birth.^[6]^ We present here a rare case of an FGCC, which extended from the right neck into the superior mediastinum, and resulted in severe tracheal compression, but completely recovered.

## 2 Case report

**Figure 1:**
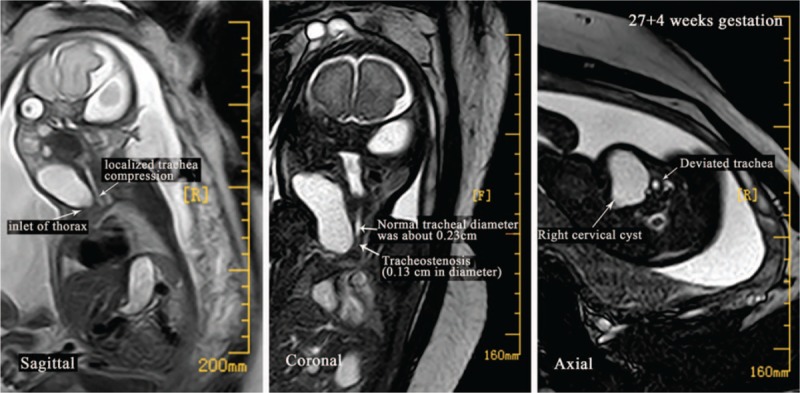
Fetal sagittal, coronal, axial MRI images at 28 weeks’ gestation (WG). MRI revealed a giant (2.2 × 4.1 × 3.6 cm) cervical cyst causing localized tracheal compression.

**Figure 2:**
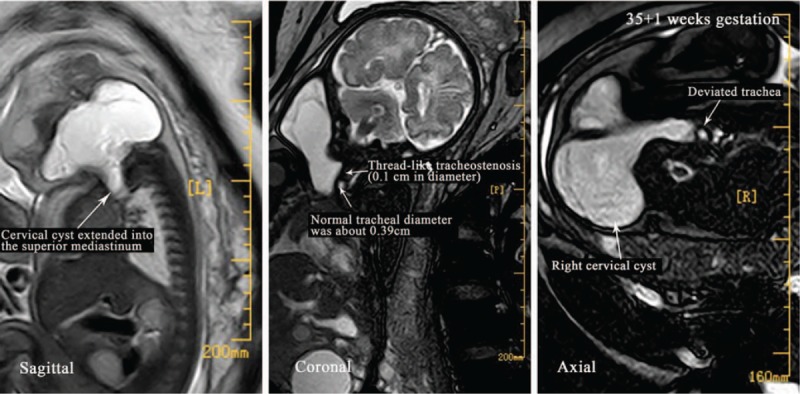
Fetal sagittal, coronal, axial MRI images at 35^+1^ weeks’ gestation (WG). MRI showed cyst growth to 3.3 × 8.2 × 7.5 cm and extended from the right neck into the superior mediastinum. The pharynx, trachea, and vessels were compressed and significantly deviated to the left. The inside diameter of the narrowest section of tracheostenosis appeared thread-like and measured only 0.1 cm.

A 31-year-old woman (gravida 6, para 1) was registered with other hospital. After initial assessment, she was closely followed at 2-week intervals until 27 weeks’ gestation (WG), which is when the first ultrasonography was performed. An FGCC was observed by ultrasonography. For further diagnosis and treatment, she was transferred to our hospital. Fetal magnetic resonance imaging (MRI) was performed at 27 + 4 WG and showed a giant (2.2 × 4.1 × 3.6 cm) cervical cyst that caused localized tracheal compression (Fig. 1). The potential for tracheal compression resulting in an inability to intubate needed to be assessed before birth to ensure safe airway management at birth. At 35 + 1 WG, fetal MRI was performed and showed that the cyst had grown to 3.3 × 8.2 × 7.5 cm and extended from the right neck into the superior mediastinum (Fig. 2). The pharynx, trachea, and vessels were compressed and greatly deviated to the left (Fig. 2). The inside diameter of the narrowest section of tracheostenosis appeared thread-like and measured only 0.1 cm (Fig. 2). These findings indicated that a fetal neck cyst may be progressive, and the likelihood of intubation failure at birth may increase. At 36 + 1 WG, 2 days before birth, cervical cyst reduction was performed under ultrasound guidance to alleviate the tracheal compression and maximize the chance of fetal survival. A total of 40 ml of lymphocyst fluid was aspirated by inserting a needle through the mother's abdomen into the uterus. Pathological analysis confirmed lymphangiomas. A histopathological diagnosis of fetal lymphangiomas was then made.

**Figure 3:**
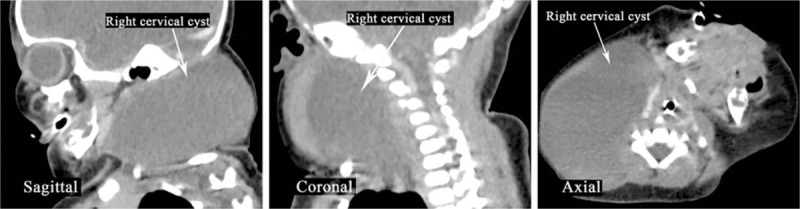
Fetal Computed tomography (CT) images after Birth. CT revealed a 4.0- × 6.1- × 4.8-cm cystic lesion without obvious strengthening in right neck; the upper part of the lesion was adjacent to the auricle and the lower part reached the right supraclavicular fossa.

At 36 + 3 WG, cesarean section was performed under general anesthesia. A female infant was delivered 4 minutes after commencing the operation. Intubation (3.5-mm tube) was performed immediately by an experienced anesthesiologist. An approximately 10- × 7- × 6-cm elastic soft mass was palpable on the right side of her neck (Fig. 3). Emergency enhanced computed tomography was performed and showed a 4.0- × 6.1- × 4.8-cm cystic lesion without obvious strengthening in the right neck. The upper part of the lesion was adjacent to the auricle and the lower part reached the right supraclavicular fossa.

**Figure 4:**
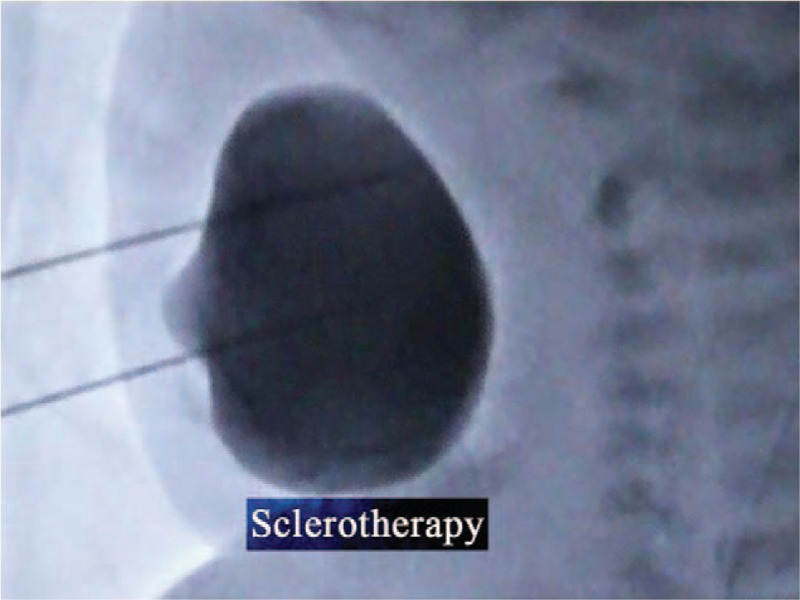
The radiography image. The radiography image showed that mixed infusion was deposited in the lesion.

Informed consent was obtained from the mother for publication of this case report and accompanying images. Intralesional sclerotherapy was preformed because surgical resection for complete removal was difficult and complications are frequent. After standard monitoring according to the American Society of Anesthesiologists guidelines, general anesthesia was then maintained with sevoflurane 3% in 2 L/minute oxygen and assisted ventilation. Spontaneous respiration was maintained at a tidal volume of 20 ml and a respiration rate of 40 to 45 bmp, and artificial assisted ventilation was performed when necessary. Any neuromuscular-blocking drugs were avoided. With strict aseptic precaution, approximately 45 ml of bloody cystic components of the lesion were aspirated with guidance by digital subtraction angiography. Sclerotherapy was then performed, and radiography showed that mixed infusion was deposited in the lesion (Fig. 4). Only 10 minutes was required to finish the operation. Extubation was performed soon after surgery. After the trachea was extubated, the neonate recovered uneventfully and was discharged 2 days postoperatively. She had no significant hoarseness, dysphagia, changes in voice quality, or dyspnea. One month later, she was treated with the sclerotherapy procedure again. After 140 days follow-up, the neonate had recovered completely (Fig. 4C).

## 3 Discussion

FGCC, which is a rare type of fetal tumor, may cause considerable fetal and perinatal effects because of compression from the mass on surrounding cervical and facial structures.^[7]^ This sometimes results in severe tracheal compression,^[8]^ which is a life-threatening event at birth. For those fetuses that survive during the antenatal period, airway obstruction may be the most life-threatening event at birth and securing airway safety has the biggest impact on immediate outcomes. The difficulty of airway control for infants with FGCC was demonstrated in a previous report.^[8]^ Of 35 fetuses who were evaluated and neck masses were diagnosed prenatally, nine died perinatally. Two fetuses died from a lack of an airway when an EXIT procedure could not be achieved, and 1 who required tracheostomy at EXTT died late because of tracheostomy complications of prematurity, respiratory failure, and sepsis.^[8]^ A newborn who does not establish breathing within 5 minutes of birth may suffer from hypoxic ischemic brain injury or even death. Therefore, congenital FGCC may require considerable planning to optimize perinatal management. Any management decisions include assessment of airway safety and consideration of short- and long-term outcomes.

Early diagnosis and treatment of FGCC is critical, and may avoid a life-threatening situation at birth. Diagnostic ultrasound is generally regarded as safe and has been used clinically in obstetrics for over 50 years. Currently, ultrasonography may play a major role in early diagnosis of FGCC. Fetal MRI is not a general screening tool for this purpose, and should only be used in occasional specific high-risk situations or used to answer specific questions raised by ultrasonography. However, FGCC is one of the appropriate fetal MRI indications. Therefore, if an FGCC is suspected by abdominal ultrasonography, fetal MRI should be performed to assess the severity of tracheal compression before birth, if feasible. This is important for subsequent clinical decisions and treatment to improve fetal short- and long-term outcomes.

Findings from our case suggested that, if treated correctly, the outcome of a fetus with FGCC is good. We suggest the following recommendations. For patients with an FGCC, airway status is critical and directly determines the fetal prognosis. When an FGCC is suspected, a multidisciplinary team comprising senior staff of the Departments of Gynecology, Obstetrics, Anesthesiology, Neonatology, Pediatrics, and Pediatric Surgery should be immediately established and discussion begun. Initial counseling should be set up immediately after sonographic diagnosis. The anesthesiologist has an important role in management of the airway. In our case, the potential for tracheal compression resulting in an inability to intubate needed to be assessed before birth to ensure safe airway management at birth. At 35 + 1 WG, fetal MRI showed that the cyst had grown to 3.3 × 8.2 × 7.5 cm and extended from the right neck into the superior mediastinum. The pharynx, trachea, and vessels were compressed and greatly deviated to the left. The inside diameter of the narrowest section of tracheostenosis appeared thread-like and measured only 0.1 cm. These findings indicated that a fetal neck cyst may be progressive, and the likelihood of intubation failure at birth may increase. This information is important to anesthesiology and obstetrics, and suggested that the EXIT procedure could not be achieved because of failure of intubation if untreated. Prenatal cervical cyst reduction under ultrasound guidance was then performed, and the fetus survived. Findings from our case suggested that an anesthesiologist should assess the risk of intubation failure at birth according to those results. If fetal severe tracheal compression is detected and it may result in inability to intubate at birth, which is a life-threatening event, prenatal cervical cyst reduction under ultrasound guidance may be an effective method, if feasible. This method could alleviate tracheal compression at birth and maximize the chance of fetal survival.

As shown in our case, intralesional sclerotherapy may be an alternative to surgical resection. During the procedure, appropriate anesthetic agents are required for an adequate depth of anesthesia. In our case, general anesthesia was performed only by an inhaled anesthetic, sevoflurane, and no neuromuscular-blocking drugs were used. The reasons for this are as follows. First, sevoflurane with rapid onset and offset, allowed easy titration of the anesthetic depth. Propofol was also titrated to maintain an adequate anesthetic depth. However, propofol causes bradycardia, hypotension, and desaturation, and its use in the neonatal period is controversial. Second, we only took 10 minutes to perform intralesional sclerotherapy. When the procedure was over, sevoflurane could be quickly expelled from the body, and extubation was performed smoothly. Intraoperative extraction of 45 ml made the mass considerably smaller. Finally, digital subtraction angiography was performed to ensure that there was no progressive bleeding in the lesion. In addition to the above-mentioned reasons, long-term endotracheal intubation required sedative drugs, which aggravate neonatal pneumonia and increase the financial burden. Therefore, extubation was performed after sclerotherapy.

In conclusion, we report a rare case of an FGCC, which extended from the right neck into the superior mediastinum, and resulted in severe tracheal compression. If an FGCC is suspected by abdominal ultrasound, fetal MRI is recommended to assess the severity of tracheal compression before birth, if feasible. An anesthesiologist should assess the risk of intubation failure at birth according to these results. If fetal severe tracheal compression is detected and it may result in inability to intubate at birth, prenatal cervical cyst reduction under ultrasound guidance may be effective for alleviating the tracheal compression at birth and maximizing the chance of fetal survival, if feasible. Improving fetal short- and long-term outcomes is important. Successful management of the airway by an anesthesiologist at birth and intralesional sclerotherapy by a pediatrician after birth are also necessary for recovery. Therefore, a well-established multidisciplinary team is also important in ensuring complete recovery of neonates with FGCC.

## Author contributions

**Data curation:** Yanming Kang.

**Investigation:** Fumin Zhao.

**Project administration:** Yushan Ma, Xuemei Lin.

**Supervision:** Xuemei Lin.

**Writing – original draft:** Yushan Ma.

**Writing – review & rditing:** Xiaoqin Jiang.
